# Formulations for Allergen Immunotherapy in Human and Veterinary Patients: New Candidates on the Horizon

**DOI:** 10.3389/fimmu.2020.01697

**Published:** 2020-08-04

**Authors:** Isabella Pali-Schöll, Douglas J. DeBoer, Claudia Alessandri, Ahmed Adel Seida, Ralf S. Mueller, Erika Jensen-Jarolim

**Affiliations:** ^1^University of Veterinary Medicine, Vienna, Austria; ^2^Institute of Pathophysiology and Allergy Research, Center of Physiology, Pathophysiology and Immunology, Medical University of Vienna, Vienna, Austria; ^3^Dermatology/Allergy Section, Department of Medical Sciences, School of Veterinary Medicine, University of Wisconsin, Madison, WI, United States; ^4^Associated Centers for Molecular Allergology (CAAM), Rome, Italy; ^5^Department of Microbiology and Immunology, Faculty of Veterinary Medicine, Cairo University, Cairo, Egypt; ^6^Centre for Clinical Veterinary Medicine, University of Munich, Munich, Germany

**Keywords:** immunotherapy, adjuvant, allergy, allergen, veterinary, human

## Abstract

Allergen immunotherapy is currently the only causal treatment for allergic diseases in human beings and animals. It aims to re-direct the immune system into a tolerogenic or desensitized state. Requirements include clinical efficacy, safety, and schedules optimizing patient or owner compliance. To achieve these goals, specific allergens can be formulated with adjuvants that prolong tissue deposition and support uptake by antigen presenting cells, and/or provide a beneficial immunomodulatory action. Here, we depict adjuvant formulations being investigated for human and veterinary allergen immunotherapy.

## Introduction

Allergen immunotherapy (AIT) is currently the only causative treatment for allergic diseases of animals and man. Subcutaneous administration of allergen extracts—with or without an aluminum hydroxide adjuvant—historically has proven efficacious for many allergic patients. However, recent studies suggest that desensitizing properties of the allergen potentially can be enhanced by alternate adjuvants or delivery systems, while maintaining freedom from adverse effects.

A number of delivery systems for AIT are currently being investigated (1–3) and applied in animal models, but rather few human or veterinary clinical studies exist. For nano- (NP) or microparticle (MP) preparations, various particulate compositions are complexed or filled with allergens (3). The particulate materials must be biocompatible (resulting in no adverse reaction) and can either be biodegradable (broken down in the organism) or non-biodegradable. Several non-biodegradable materials tested as delivery systems for allergens *in vitro* as well as in animal models, such as dendromers/dendrosomes (4), polyethylenimine (5), polypropylene sulfide (6), multiwalled carbon nanotubes (7), gold nanoparticles (8), or fullerenes (9) have been comprehensively reviewed (2). However, their fate in the organism is not absolutely clear and thus must be carefully studied.

We selected here the most promising novel AIT formulations, encompassing both modifications of allergen and inclusion of immunomodulators, and describe their performance in human and veterinary trials.

## Formulations With Vehicles That Protect Immunogenicity

For an allergen-specific and prolonged effect of AIT, allergens must reach the immune system in a recognizable form, and be released from any carrier in an optimal, perhaps gradual manner. Thus, one general approach to enhance response to AIT is to protect allergens from degradation, and/or ensure optimal release by packing them into resistant carrier materials.

### Methylmethacrylate Coating

Grass pollen allergen was coated with a co-polymer of methacrylic acid and methylmethacrylate, called Eudragit L-100®, to protect against gastric degradation, and administered orally to Guinea pigs (10). The secondary antibody response was greater than with an aqueous solution of the allergen. An encapsulated ragweed allergen extract given to people with hay fever led to an increase of anti-ragweed IgG antibodies, a dampened increase of IgE antibodies, and decreased symptom-medication scores without systemic reactions (11).

### Plant Cell-Wall Fusion Proteins

Plant cell-expressed or complexed allergen proteins delivered orally are protected from gastric acid and enzymatic degradation, but are then digested by gut microbes in the colon and release the allergens to the immune system (12). Transgenic rice expressing the major house dust mite (HDM) allergen Der p 1 was developed as an edible AIT product (13). Several other proteins have been used in this fashion to induce tolerance in mice. After oral prophylactic administration of transgenic rice expressing modified Japanese cedar pollen allergens Cry j 1 and Cry j 2 (14, 15) to BALB/c mice or HDM allergen Der p 2 in transgenic tobacco in a murine asthma model (16), a decreased allergic response was uniformly seen. Chemically modified ragweed pollen shells fed to BALB/c mice were incorporated in the subepithelial tissue (17). In addition, bone-marrow derived macrophages and dendritic cells cultured with this pollen increased expression of CD40, CD80, CD86, and MHC class II molecules and secreted proinflammatory cytokines TNF-alpha and IL1-beta. Such studies have not been performed in human or veterinary patients.

### Polyanhydrides

Particles made of amphiphilic polyanhydrides are biodegradable and show a favorable safety profile. Poly[methyl vinyl ether-co-maleic anhydride] (Gantrez® AN 119) has been investigated in mouse models for oral immunotherapy against peanut allergy (18–20), cashew nut allergy (21), and *Lolium perenne* pollen allergy (22). Three doses of nanoparticle-coated peanut allergens were able of protecting CD1 mice against severe anaphylaxis induced by a peanut challenge (18). Similarly, in CD1 mice presensitized to peanut, AIT with nanoparticle-encapsulated peanut allergen was associated with significantly lower concentrations of mMCPT-1, and an increased survival rate after challenge, compared to AIT with free peanut extract (20). Similar results were seen with allergens of *L. perenne* combined with Gantrez nanoparticles and LPS of *Brucella ovis* (22). Oral administration of cashew nut-loaded nanoparticles to BALB/c mice led to a decrease in splenic Th2 cytokines, and an enhancement of pro-Th1 and regulatory cytokines with an increased expansion of T regulatory cells compared to mice immunized with free allergens (21). Despite promising results in these murine models, no published studies in human or veterinary patients exist.

Evidence thus indicates that formulations protecting the allergens are beneficial, and show Th1- and Treg-inducing capacity.

## Allergens Administered With Novel Adjuvants

A different approach incorporates adjuvants along with the allergen, with the goal of enhancing a desirable, non-allergic immune response, optimally counteracting an allergy-immune milieu.

### Monophosphoryl Lipid A (MPLA)

Monophosphoryl lipid A (MPLA) is a compound derived from Gram-negative bacteria and effectively applied in human allergic patients since 1975 (23). *In vitro* studies indicate that it may also induce the secretion of Th1 cytokines from equine cells, thus making it a candidate for the treatment of insect bite hypersensitivity (IBH) (24). Twelve healthy Icelandic horses were immunized with *Culicoides nubeculosus* allergens adjuvanted with MPLA plus alum, or alum alone (25). When their peripheral blood mononuclear cells (PBMCs) were stimulated, the MPLA/alum-immunized horses produced more IFN-gamma and IL-10, both preferable in allergy.

### Gelatin-CpG-ODN

Gelatin particles combined with CpG-ODN (GbpCpG) are among the few preparations already studied in veterinary allergy patients, including canine atopic dermatitis (26, 27) and equine recurrent airway obstruction (RAO, an analog of human asthma) (28–30). Uptake of these particles by canine PBMCs could be demonstrated with confocal laser scanning microscopy, and an increase of IL-10 secretion could be shown when cells were incubated with GbpCpG compared to CpG-ODN alone (27). Atopic dogs improved clinically after subcutaneous administration of GbpCpG, while their IL-4 expression decreased (26). Bronchoalveolar lavage cells from RAO horses were incubated with different CpG-ODN sequences; IL-10 and IFN-gamma release was increased, while IL-4 decreased (30). When nebulized with a gelatin nanoparticle-based CpG-ODN formulation, horses with RAO improved clinically and the IL-10 concentration increased in their bronchoalveolar lavage fluid (28). In a subsequent placebo-controlled trial, this treatment caused a persistent decrease of allergic clinical variables in horses treated with nebulized GbpCpG (29). A later study described lyophylisation of GbpCpG facilitating its storage and use (31).

### Triacedimannose (TADM)

Incubation of the synthetic trivalent glycocluster TADM with birch-stimulated PBMC of allergic rhinitis patients suppressed the production of all Th2-type cytokines (32). TADM suppressed IgE production and enhanced IFN-gamma production in a mouse model of OVA-induced allergic asthma (32). Intranasal application of TADM and timothy grass pollen extract to sensitized BALB/c mice led to a much greater decrease in lymphocyte and eosinophil counts in blood, BALF, and lung biopsies compared to CpG-ODN and MPLA, and (in contrast to CpG-ODN alone) did not increase neutrophil counts (33).

### Polysaccharide Polymers

Carbohydrate-based particles complexed with Phl p 5 grass pollen allergen or cat allergen Fel d 1 were successfully used in several studies of AIT in mice (34–37).

The polyaminosaccharide chitosan (poly-D-glucosamine) is approved for use in human wound healing, but is not yet evaluated for AIT. Chitosan particles were used with ovalbumin as a mucoadhesive to promote uptake by oromucosal dendritic cells *in vitro* (38), and also with allergens from HDM and peanut in mouse models to augment AIT (39–41).

Other polysaccharides used for preparation of particulate delivery systems are dextran, alginate, starch, and cellulose derivates. Amylopectin-based microparticles were formulated with Bet v 1 from birch pollen for sublingual treatment of allergic mice (42). Mannan-dextran-maltodextrin covalently attached to OVA and papain were intradermally injected into BALB/c mice, leading to elevated humoral immune responses, and an IgE-to-IgG-shift (43). Another potentially useful polysaccharide is pullulan, a polysaccharide which, coupled to HDM allergen Der p 2, and administered to dogs, effectively reduced clinical signs of atopic dermatitis (44). Carbohydrate-modified ultrafine ceramic-core based nanoparticles, so-called aquasomes, are not biodegradable, and have been applied in the mouse model with ovalbumin (OVA) as model allergen preparation for intradermal application (45).

### Heat-Labile Toxin (LT) From *E. coli*

A patch delivery system for birch pollen allergen rBet v 1 with and without heat-labile toxin (LT) from *Escherichia coli* was compared to subcutaneus alum-adsorbed rBet v 1 in a guinea pig model (46). Only the rBet v 1-LT was able to induce allergen-specific blocking IgG antibodies comparable to subcutaneous immunization.

### Miscellaneous Particulate Formulations

Strontium-doped hydroxyapatite porous spheres (SHAS) have been used with OVA subcutaneously in a mouse model and led to a sustained stimulation of both CD4+ and CD8+ T cells (47). AIT with SHAS-OVA showed a higher efficacy as assessed by symptom scores compared to soluble OVA. This approach was not tested clinically in human or veterinary patients.

Poly(epsilon-caprolactone; PCL) is a biocompatible adjuvant, and in mice sensitized to OVA led to lower IgE, fewer anaphylactic reactions, and higher survival rate compared to alum-adjuvant treated animals (48). Studies in human and veterinary patients are lacking.

Modified difunctional water-soluble PEG dimethacrylate (PEG-acetal-DMA) macromonomers have cleavable acetal units (49), and when those were filled with allergen (OVA, grass pollen allergen, HDM allergen) and encapsulated into liposomes, they could avoid IgE-dependent activation of basophils *in vitro*, but were taken up by dendritic cells (50).

Poly-glutamic acid particles (PGA) were used with *Phleum pretense* pollen extract *in vitro* and increased allergen-specific IL-10 production and proliferation of autologous CD4+ memory T cells (51). Other investigators have shown that PGA *per se* is an allergen in fermented soybeans, which causes hypersensitivity reactions and even late-onset anaphylaxis (52–54). To the authors' knowledge, there are no studies evaluating PGA in animals.

Protamine-based nanoparticles are biodegradable and biocompatible arginine-rich peptides. When complexed with Ara h 2 from peanut and CpG-ODN, they could counteract a Th2-dominated allergen-induced immune response in mice (55). A combination of liposomes with protamine and DNA was also proven effective in combating *Chenopodium album* allergy in a mouse model (56). At this point, there are no published clinical studies with protamine-based nanoparticles.

Mesoporous silica nanoparticles were successfully used in allergy models (57) with HDM allergen Der f 2 for subcutaneous prophylactic treatment of mice (58). However, when applied epicutaneously with mite extract in the form of agglomerates, they induced AD-like skin lesions and promoted IgE-responses (59). Studies in human and veterinary patients are lacking.

Taken together, many of novel adjuvants have shown Th1-promoting capacity *in vitro* and *in vivo* in murine models and even veterinary patient studies for horses and dogs. They were capable of counter-acting IgE, inducing preferentially IFN-γ, and/or IL-10 and also resulting in reduced symptom scores, being more effective than their non-adjuvanted controls.

## Allergens Coupled to Immunomodulators

Efforts have also been made to enhance an overall shift in the immune response away from Th2, while at the same time presenting the offending allergen. Some approaches incorporate elements that can redirect the overall immune response from an allergy-prone Th2-IgE-milieu to a more Th1-IgG-dominated reponse.

### Modified Adenine Conjugates

Der p 2 allergen bound to 8-OH-modified adenine (nDer p2-Conj) forms an allergen-TLR7 agonist conjugate. When injected subcutaneously, it reduces allergen challenge-induced murine airway inflammation (60–62), triggers TLR7, redirects allergen-specific Th2 responses, and promotes a Th1 response as well as an increase in IL-10 with prolonged effects.

### Mannan-Modified Allergens and Allergoids

Mannan preparations, alone or allergen-conjugated, appear capable of downregulating IgE responses. Konjac glucomannan (Amorphophallus konjac) fed to BALB/c mice suppressed IgE class switching in B cells and inhibited Th1 and Th2 responses (63). It also suppressed IgE production and clinical signs in a mouse model of allergic rhinitis (64). Administration of neoglycocomplexes of mannan with ovalbumin and papain to sensitized mice led to a class switch from IgE to IgG, and to a decrease in basophil degranulation *in vitro* (43).

Polymerized allergoids have been coupled to non-oxidized mannan from *Saccharomyces cerevisae* (PM-allergoids); this is one of the few modified allergen preparations that has been studied in dogs (65). Dendritic cells capture PM-allergoids better than native allergens and enhance Th1/Treg cell responses upon subcutaneous or sublingual administration (66, 67). Interestingly, the addition of alum may impair their tolerogenic properties (68).

### DNA Engineered Hybrids With Copolymers

Hybrid allergen molecules are obtained by combining the epitopes of several allergens. Subsequently, their immunogenicity can be enhanced by coupling with copolymers. Engineered hybrids expressing the major allergens from *Parietaria* pollen allergens Par j 1 and Par j 2 were prepared as nanoaggregated copolymers with poly (hydroxyethyl)-aspartamide (PHEA). They are biodegradable, water-soluble and showed low cytotoxicity, no effect on hemolysis, and no non-specific activation of basophils. Basophil activation properties were, however, maintained in cells from *Parietaria*-allergic subjects, indicating preserved crosslinking capability of the hybrid allergen (69). No *in vivo* studies have been reported with this preparation.

### Allergen Linked to CpG Oligodeoxynucleotides (CpG-ODN)

CpG-ODN are short, single-stranded synthetic DNA molecules with immunostimulatory properties that induce a Th1-based immune response (70), which prevents Th2-mediated hypersensitivity in mouse models of allergic diseases such as allergic rhinitis (71), asthma (72), conjunctivitis (73), and anaphylactic shock (74). Purified Amb a 1 from *Ambrosia artemisiifolia* pollen linked to CpG-ODN was successfully tested subcutaneously in humans and resulted in a shift from Th2 to Th1 with an increase of IFN-γ and a decrease in IL-5, proving suitable as an agent for immune redirection in immediate hypersensitivity diseases (75).

### Siderophore-Bound Iron or Retinoic Acid as Immunomodulatory Ligands

Bos d 5 cow milk allergen is capable of binding iron via siderophores. The immunomodulatory properties of iron-bound allergen were tested *in vitro* with human PBMC (76). The empty *apo*-form of Bos d 5 increased CD4+ cells, IL-13, and IFN-gamma, whereas the complexed *holo*-form decreased CD4+ cells and induced apopotosis. Similarly, only the *apo*-form of birch pollen allergen Bet v 1 led to an increase in IL-13, while IFN-gamma was increased with both formulations when incubated with human PBMC (77). Accordingly, spiking of Bet v 1 or Bos d 5 with iron may be an effective approach to improve the efficacy of AIT against birch pollen and cow milk allergy, respectively (76, 77).

The major allergen Bos d 5 was also complexed with the vitamin A metabolite retinoic acid (78). IgE binding was not influenced, but PBMCs from healthy people stimulated with the complex led to a decrease of CD4+ T cells as well as IFN-gamma, IL-13 and IL-10, although induction of CD4+CD25+Foxp3+ regulatory T cells was not seen (79). In contrast to *apo*-Bos d 5, a highly allergenic molecule, *holo*-Bos d 5 thus seems to have reduced immunogenicity.

### Expression of Allergens by Bacterial Vectors

*Streptococcus thermophilus* (ST) expressing rBet v 1 was evaluated in a mouse model (80). BALB/c mice were sensitized with rBet v 1 and then treated orally with either ST, ST and rBet v 1, or ST expressing rBet v 1. After aerosol challenge, T regulatory cells, IL-10, and IFN-gamma were increased with the expressed-allergen preparation; bronchial eosinophilia, allergen-induced IL-4, and the rBet v 1-specific IgE/IgG2 ratio were decreased, indicating a shift from Th2 to Th1 and Treg immune responses (80).

Profilin (Che a 2), the major allergen of *C. album*, was expressed in *Lactobacillus lactis*, and was bound by human anti-profilin IgE (81). However, bacterial survival was greatly reduced with low pH and simluated gastric and intestinal juices. Oral vaccination with recombinant *Lactobacillus plantarum* expressing the Japanese Cedar pollen allergen Cry j 1 led to a suppressed allergen-specific IgE response and decreased nasal symptoms in a murine model of allergic rhinitis (82).

### Allergens Conjugated to Bacterial Products

Bacterial surface S-layer proteins (SLPs) are two-dimensional crystalline arrays of glycoprotein subunits present on the outermost layer of many bacteria, and have strong adjuvant properties. Conjugating recombinant allergens with SLPs leads to strongly reduced IgE-binding activity and promotes the induction of allergen-specific Th0/1 cells and regulatory T cells. This type of allergen modification has been attempted with inhalant allergens (83). Subsequently, bacterial S-layers have been studied as carriers for peanut allergen-derived peptides (84, 85). A fusion protein of an Ara h 2-derived protein and an S-layer protein was recognized by Ara h 2-specific IgE of human patients but was not able to degranulate sensitized rat basophils *in vitro* (84). The A20, tumor necrosis factor-induced protein 3 (TNFAIP3), is a ubiquitin-modifying protein playing a defensive role in the pathogenesis of allergic diseases. A DNA vaccine coexpressing Der p 2 and ubiquitin A20 encapsulated into nanoparticles used intranasally in a murine model of allergic rhinitis was able to inhibit allergen-specific IgE, IL-4, IL-10, and IL-17 secretion and to increase IgG1, IgG2a, and IFN-γ (86, 87).

A genetically engineered inhalative cholera toxin B subunit/allergen fusion molecule, CTB-Bet v 1, was shown to improve the immunomodulatory capacity of the mucosal delivery system better than chemically coupled products (88).

Overall, the concept of redirecting the immune response from a Th2 to a Th1-bias as part of AIT has promise. However, most immunomodulatory components—except for CpG-ODN—have been tested in murine models only, and need to be further tested in human and veterinary patients.

## Combination and Miscellaneous Approaches

Several formulations combine the enhancing and modulating effect on the immune response, in parallel to protecting the antigen from degradation or digestion, and further releasing it in a delayed manner. Different particulate formulations together with immune-cell targeting substances have been used for these attempts.

### Liposomes

Liposomes are bilayers of phospholipids, forming vesicles which can transport aqueous substances inside. They are biocompatible, biodegradable, and can be co-formulated with oligomannose coats, a preparation that was tested in human HDM-allergic asthma patients (89). Mouse models were used to study the efficacy of liposomes in treating allergy against Japanese cedar pollen (90), HDM (91), cat (92), OVA (93), or cockroach (94). Lipid nanoparticles together with *Parietaria* allergen Par j 2 were characterized biochemically and biophysically (95). Liposome complexes with CpG-DNA and individual allergen extracts were used intradermally for treatment of canine atopic dermatitis after failure of conventional AIT (96). Pruritus improved and IL-4 production decreased with treatment (96). Chronic rhinitis in adult cats could be treated with feline IL-2-filled liposomes plus DNA, although a Th2 bias could not be identified in those cats (97). Liposomes with HDM allergens Der p 1 or Der p 2 reduced clinical and medication scores, skin test responses, and bronchial challenge responses in asthmatic patients (89).

### Poly-Lactic-Co-Glycolic Acid Particles (PLGA, PLG, PLA)

These polyesters are approved for use in people as absorbable surgical suture. In mouse models for birch allergy, they were successfully administered subcutaneously with Bet v 1 (98, 99). In addition, PLGA-microparticles were used orally with different plant lectins e.g., *Aleura aurantia* lectin, wheat-germ agglutinin or *Ulex europaeus*-I, or *Vibrio cholerae* neuraminidase to target mucosal cells for enhanced uptake (100–103). Other allergens used with PLGA-particles in animal models via different routes are the *Chenopodium* allergen rChe a 3, as sublingual immunotherapy in a mouse model of allergic rhinitis (104, 105), Ole e 1 from olive pollen or T cell epitopes thereof for intranasal prevention (106, 107), bee venom allergen PLA2 (108), pollen-profilin from palm *Caryota mitis* (109), Der p 2 from HDM (110), peanut extract (111), or beta-lactoglobulin from milk whey (112). PLGA locally induced a regulatory T cell response via the incorporated mediator substances TGF-beta-1, rapamycin, and IL-2 to prevent a subsequent contact dermatitis reaction (113). In addition to complexing PLGA-particles with allergens, PLGA were complexed with immune-modulating substances such as CpG-ODN for allergy and asthma prevention (114) and with Der p 2-A20 DNA in allergic rhinitis (87) in mouse models. There are no studies in companion animals with PLGA.

### Virus-Like Particles (VLP)

Virus-like particles are used as carriers for allergens, or without antigen for antigen-independent immunomodulation (115). Particles consisting of bacteriophage coat proteins and a TLR-9 agonist, but without allergen, were injected into HDM-allergic patients and led to lower symptom-medication scores, higher quality of life and better allergen tolerance (116). A second study with A-type CpG-ODN and HDM-extract showed similar results; allergen-specific IgG increased as well (117). Recently, equine IBH was safely treated with IL-5-linked VLP made from cucumber mosaic virus to induce auto-antibodies against IL-5 (118–121). Clinical signs of treated horses improved and their eosinophilia was decreased compared to controls. The same principle was used successfully with IL-31-linked VLP for treatment of IBH in horses and for atopic dermatitis in dogs (122, 123). A very interesting approach is the immunization of cats with Fel d 1-VLPs (HypoCat^TM^) to induce a neutralizing antibody response in the animal against its own Fel d 1-protein for protection of humans against cat allergy (124, 125). In BALB/c mice, adeno-associated VLP were also tested with an OVA-derived B cell epitope (126), with Art v 1 from mugwort (127) and with peanut allergens Ara h 1 and Ara h 2 (128). Fel d 1 displayed on VLPs failed to induce human mast cell activation *in vitro* (129). The peptide HDM allergen Der p 1 was coupled to a virus-like particle derived from a bacteriophage and injected in healthy volunteers. Significant IgG responses against the allergen were observed and the vaccine was well-tolerated (130).

### *Aleuria Aurantia* Lectin (AAL)

AAL is derived from the edible orange peel mushroom *A. aurantia*. When birch pollen-sensitized BALB/c mice were fed with birch pollen-AAL-microspheres, the birch pollen-specific IgG2a, but not IgG1 or IgE increased, as well as IFN-gamma, IL-10, and IL-4 (101). Oral administration of birch pollen-AAL-MS led to an IgG2 antibody response in naive BALB/c mice (102). AAL microspheres may have the potential to serve as a vehicle and adjuvant for oral immunotherapy, potentially stimulating specific mucosal immune responses via M-cell targeting (100).

### Wheat Germ Agglutinin (WGA)

Birch-pollen allergens were entrapped in poly(D,L-lactic-co-glycolic acid) microspheres, further coated with WGA to target enterocytes used for oral immunotherapy of type I allergy to protect allergens from digestion and to support intestinal uptake (131). The antigenicity of the birch pollen was maintained at ~60% even after 2 h of simulated gastric digestion, and allergen-specific IgG serum concentrations increased in BALB/c mice fed with the WGA-birch pollen-microspheres (131).

With these approaches, VLP, liposomes, and PLGA particles seem to have promise, and are already tested in human, canine, feline, and equine patients.

## Summary

Allergen immunotherapy is the only treatment for allergic diseases that is truly causal and modifies the course of the ongoing disease. As this review discusses, many dozens attempts have been made to identify adjuvants, immunomodulators, physical packaging, conjugates, and combinations of the above to modify allergenic proteins, making them safer, and more efficacious in AIT. Many of the formulations have scarcely progressed beyond *in vitro* studies, though some show great promise in rodent models. Our task is now to select the most promising candidates, and carry them forward into preclinical studies that can more carefully predict which will translate into clinical benefit. Because many human allergic diseases are found nearly identically in animals, veterinary studies could serve as an elegant precursor to the same investigations in human patients.

## Author Contributions

IP-S and EJ-J designed and drafted the manuscript, wrote abstract and introduction. IP-S wrote part on particulate delivery systems. CA worked on allergen modifications. AS contributed immune modulators/activators. RM worked on all the parts completing results and references. DD wrote summary and edited, formatted and finalized MS. All authors contributed to the article and approved the submitted version.

## Conflict of Interest

EJ-J declares inventorship in patents on allergen immunotherapy formulation with Biomedical International R+D, Vienna, Austria, of which she is shareholder, and is business partner of Bencard Allergie, Germany as well as AllergyTherapeutics, UK. Within the last 5 years, RM has been a consultant, lecturer, or has received financial support for studies from Artuvet, Greer Laboratories, Heska Laboratories, Nextmune, and Synlab. The remaining authors declare that the research was conducted in the absence of any commercial or financial relationships that could be construed as a potential conflict of interest.

## Abbreviations

AIT: allergen immunotherapy
FDA: U.S. Food and Drug Administration
HDM: house dust mite
MP: microparticle
MPLA: monophosphoryl lipid A
NP: nanoparticle
ODN: oligodeoxynucelotides
OVA: ovalbumin
PEG: polyethylene glycol
PGA: poly-glutamic acid
PHEA: poly(hydroxyethyl)-aspartamide
PLGA: poly-lactic-co-glycolic acid
TLR: Toll-like receptor
VLP: virus-like particles
RAO: recurrent airway obstruction
IBH: insect bite hypersensitivity
PBMC: peripheral blood mononuclear cells
WGA: wheat germ agglutinin
SLP: S-layer protein
LT: heat-labile toxin
SHAS: Strontium-doped hydroxyapatite porous spheres
TADM: Triacedimannose.
